# Seizure-Induced *Arc* mRNA Expression Thresholds in Rat Hippocampus and Perirhinal Cortex

**DOI:** 10.3389/fnsys.2018.00053

**Published:** 2018-11-01

**Authors:** Monica K. Chawla, Daniel T. Gray, Christie Nguyen, Harshaan Dhaliwal, Marc Zempare, Hiroyuki Okuno, Matthew J. Huentelman, Carol A. Barnes

**Affiliations:** ^1^Evelyn F. McKnight Brain Institute, University of Arizona, Tucson, AZ, United States; ^2^ARL Division of Neural Systems, Memory and Aging, University of Arizona, Tucson, AZ, United States; ^3^Medical Innovation Center, Kyoto Graduate School of Medicine, Kyoto University, Kyoto, Japan; ^4^Translational Genomics Research Institute, Neurogenomics Division, Phoenix, AZ, United States; ^5^Departments of Psychology, Neurology, and Neuroscience, University of Arizona, Tucson, AZ, United States

**Keywords:** *in situ* hybridization, calcium plateau potentials, seizures, confocal microscopy, immediate-early genes

## Abstract

Immediate-early genes (IEGs) are rapidly and transiently induced following excitatory neuronal activity including maximal electroconvulsive shock treatment (ECT). The rapid RNA response can be blocked by the sodium channel antagonist tetrodotoxin (TTX), without blocking seizures, indicating a role for electrical stimulation in electroconvulsive shock-induced mRNA responses. In behaving animals, *Arc* mRNA is selectively transcribed following patterned neuronal activity and rapidly trafficked to dendrites where it preferentially accumulates at active synapses for local translation. Here we examined whether there is a relationship between the current intensities that elicit seizures and the threshold for *Arc* mRNA transcription in the rat hippocampus and perirhinal cortex (PRC). Animals received ECT of varying current intensities (0, 20, 40 65, 77 and 85 mA) and were sacrificed 5 min later. While significantly more CA1, CA3 and perirhinal pyramidal cells expressed *Arc* at the lowest stimulus intensity compared to granule cells, there was an abrupt threshold transition that occurred in all four regions at 77 mA. This precise threshold for *Arc* expression in all temporal lobe neurons examined may involve regulation of the calcium-dependent mechanisms that are upstream to activity-dependent IEG transcription.

## Introduction

Electroconvulsive shock treatment (ECT) has been used in the treatment of psychiatric depression or mood disorders where pharmacotherapy has failed. Although the mechanisms by which ECT has its therapeutic effects are largely unknown, it is clear that inducing local seizure-like activity in the brain can alter brain chemistry, connectivity and physiology enough to reverse symptoms of certain mental illnesses (Singh and Kar, 2017). Among its many effects on the nervous system, ECT has been shown to increase the expression of several neurotrophic factors critical to synaptic plasticity, nerve growth, cell repair and survival (Zhang et al., 2009; Hu et al., 2010; Brunoni et al., 2014). In rodents, for example, ECT increases the expression of BDNF and its primary receptor (tropomyosin-related kinase B). Both proteins are known to be critical for short- and long-term potentiation (Nibuya et al., 2002; Altar et al., 2003; Lu et al., 2014; Leal et al., 2017).

Neurotrophic factors like BDNF are known to exert many of their intracellular effects through interactions with a number of the immediate early genes that are transiently expressed following synaptic activity, including ECT (Lyford et al., 1995; Bramham et al., 2008). Arc/Arg3.1 is an immediate early gene that has been shown to create postsynaptic trafficking endosomes through which AMPA receptor densities at the synapse are regulated, and is consequently considered a cellular marker of synaptic plasticity (Guzowski et al., 2001; Chowdhury et al., 2006; Shepherd et al., 2006; Bramham et al., 2008; Okuno et al., 2012). Arc/Arg3.1 is strongly induced in the rodent hippocampus and cortex within 5 min following ECT and remains elevated for 8 h, prior to returning to baseline levels within 24 h (Lyford et al., 1995; Wallace et al., 1998). During spatial navigation, location-specific firing during theta oscillations provide the necessary stimulation to drive Arc/Arg3.1 expression in the hippocampus, and there are numerous reports that demonstrate increased Arc/Arg3.1 expression following periods of behavioral exploration (Guzowski et al., 1999; Bramham et al., 2008; Hartzell et al., 2013; Chawla et al., 2018).

Many of the neurotrophic factors that elicit immediate-early genes (IEGs) transcriptional responses are regulated by calcium levels, particularly from influxes through L-type calcium channels and NMDA receptors (Tabuchi et al., 2000). It has been shown that different levels of physiological activity result in different calcium dynamics within the cell and also distinct patterns of immediate early gene responses (Dolmetsch et al., 2001; Takasu et al., 2002; Park and Poo, 2013). For example, dendritic calcium plateau potentials in CA1 pyramidal cells result from a certain level of depolarization elicited by temporally precise coincident input from CA3 and entorhinal cortical afferents. These potentials have been shown to precede the development of place-specific firing in mice that traverse a track in virtual reality (Kamondi et al., 1998; Jarsky et al., 2005; Sjöström and Häusser, 2006; Tsay et al., 2007; Takahashi and Magee, 2009; Bittner et al., 2015). Because Arc/Arg3.1 transcription is known to be calcium-dependent, these plateau potentials may contribute to the regulation of Arc/Arg3.1 behavior-driven gene expression. This suggestion predicts that Arc gene expression may show a physiological induction threshold since plateau potentials emerge only following specific stimulation patterns. The present study was undertaken to systematically investigate the question of whether there is an amplitude threshold for *Arc* expression following ECS treatment.

## Materials and Methods

Young F344 rats (5–6 months old, Harlan Sprague-Dawley, Indianapolis, IN, USA) were used in accordance with NIH guidelines and Animal Care and Use Committee at the University of Arizona. Animals were individually caged with free access to food and water. Rats (*n* = 21) were assigned to one of the six groups. Five groups received ECT of varying intensity (20, 40 65, 77 and 85 mA) using a UGO Basile ECT unit (Via Giuseppe Di Vittorio 2-21036, Gemonio-Varese, Italy). A sixth group did not receive any shock and were sacrificed directly from their home cages. This group will be referred to as caged controls. Briefly, rats were brought from the colony room in a flower pot wrapped in a towel, two leads equipped with ear clips from the ECT unit were soaked in saline and were attached to the animal’s ears prior to giving them the shock. Animals were not restrained during the application of current but an experimenter was ready to hold the rat if he jumped out of the pot following shock treatment. The duration of shock was 1 s, at 100 Hz with a 0.5 ms square wave pulse. All animals exhibited signs of behavioral seizures. We conducted a more detailed seizure assessment using seven additional animals that received different current intensities. An ECT sham was implemented by applying the saline-soaked ear clips to the ears without passing any current. This animal did not exhibit any noticeable behavioral change and moved around the pot freely, exploring and grooming. At the 10-mA current intensity the rat showed an abrupt movement of the head at the onset of ECT. Once the shock ended, the animal appeared to be slightly dazed; however, returned to normal after 2 min. At the 20-mA current intensity the rat exhibited an abrupt movement of the head, a rapid respiratory rate increase and remained motionless in the pot for 1 min before moving again. At the 40-mA current intensity, the animal experienced head movement and forelimb clonus during ECT. This was followed by an increase in 144 respiratory rate, ear twitching, and remained motionless for 2 min. At the 65-mA current intensity there was head movement and rearing with forelimb clonus for approximately 1 min, followed by clenching of the hind limbs for another minute. Subsequently the rat fell to his side. At the 77- and 85-mA current intensity, rats experienced rearing and falling with forelimb clonus and hind limb extension with convulsion for ~2 min.

### Brain Extraction and Dissection

Five minutes following treatment, rats were anesthetized with 5% isoflurane and decapitated with a rodent guillotine. Brains were rapidly removed, hemisected with the right hemisphere quickly frozen in isopentane cooled over an ethanol/dry ice bath and stored at −80°C until sectioning for *in situ* hybridization. Twenty micron thick sections were cut after blocking the hemi-brains in optimal cutting temperature (OCT) compound such that all the experimental groups and negative controls (caged) were included on the same slide to minimize technical variability. The sections were cut and thaw-mounted onto frosted slides and stored at −80°C until fluorescence *in situ* hybridization was performed.

### Fluorescence *in situ* Hybridization

Riboprobes were generated from the full length Arc cDNA (~3 K bp in length, described in Lyford et al. (1995) using a commercial RNA transcription kit (Maxiscript; Ambion, Austin, TX, USA) and RNA labeling nucleotide mix containing digoxigenin-tagged UTP (Roche Molecular Biochemicals, Nutley, NJ, USA). Fluorescence *in situ* hybridization was performed as described previously (Chawla et al., 2005). Briefly, slides containing the sections were thawed to room temperature, fixed with freshly prepared buffered paraformaldehyde (4%) and treated with 0.5% acetic anhydride/1.5% triethanolamine for 10 min. Incubated in methanol and acetone (1:1) for 5 min and equilibrated in 2× SSC. Sections were incubated with 100 μl 1× prehybridization buffer (Sigma, St. Louis, MS, USA) for 30 min at room temperature. Approximately 100 ng of riboprobe was diluted in 1× hybridization buffer (Amersham, Piscataway, NJ, USA), heat denatured at 90°C, chilled on ice and applied to each section. A coverslip was placed on each slide and incubated overnight at 56°C. Post hybridization washes started with 2× SSC, increased in stringency to 0.5× SSC at 56°C. RNase A (10 μg/ml) at 37°C was used to dissociate any single stranded RNA. After quenching the endogenous peroxidases with 2% H_2_O_2,_ slides were blocked with NEN blocking agent (Perkin Elmer, Boston, MA, USA) and incubated with an anti-digoxigenin Ab conjugated with HRP (Roche Molecular Biochemicals, Nutley, NJ, USA) overnight at 4°C. Slides were washed with Tris-buffered saline containing 0.05% Tween-20 and the HRP-antibody conjugate was detected using a Cyanine-3 (CY3) tyramide signal amplification kit (Perkin Elmer, Boston, MA, USA). Coverslips were applied to the slides after counterstaining with DAPI contained in a small amount of Vectashield antifade media (Vector Labs, Burlingame, CA, USA) and sealed with nail polish.

### Confocal Microscopy and Cellular Analysis

Confocal images were acquired using a Leica SP5 microscope equipped with 405 nm and 543 nm lasers, with a 10× dry lens or a 40× oil immersion lens. Laser settings, detector gain and offsets were kept constant after initial optimization for each slide. Overlapping images were obtained of entire dentate gyrus (DG), and *Arc* mRNA-positive granule cells were counted. Since animals were sacrificed after 5 min *Arc* mRNA was present in the nucleus as foci only. An estimate of total number of granule cells was used to obtain the percentage of total *Arc*-positive cells in the 0, 20 and 40 and 65 mA stimulus conditions. For the 77 and 85 mA condition, negative cells were counted to derive the percentage of *Arc* expression. For the CA1 subregion, images were obtained of proximal, distal and medial CA1 and for the CA3 subregion all the pyramidal cells were imaged. Areas of analysis from the CA1, CA3 or area 35 of the perirhinal cortex (PRC) were optically sectioned at ~0.75 μm in the z-plane. Three different CA1or CA3 subregions per animal were imaged in triplicate. For area 35 of the PRC, all layers were imaged in triplicate. *Arc* mRNA-positive cells were counted by an experimenter blind to the conditions using Fiji app in ImageJ. The entire DG was imaged from two to three slides per animal using the 10× objective. The area of the granule cell layer and the total number of neurons was assessed in each reconstructed flat image. The area was used to estimate the total number of neurons using a correction factor that represented the total neurons per square micron. This factor was derived from 92 Z-stacks from 10 different rats collected at 40× magnification for the dorsal hippocampus. The total number of neurons/stack was counted and the area of the granule cell layer (in μm^2^) from the middle plane was calculated (Chawla et al., 2005; Ramírez-Amaya et al., 2005). Utilizing this factor, the percent of neurons with *Arc* mRNA in the DG of each rat was calculated according to the following formula:

$$100*\text{p/}\left({\text{A}}_{\text{p}}*(\text{N}/\text{A})\right),$$

where:

p = the number of *Arc* (+) neurons in a given reconstructed flat image,
A_p_ = the area (in μm^2^) of the DG, as measured from the reconstructed flat image,
N = the total number of cells from all 40× Z-stacks,
A = the total area (in μm^2^) of the DG from the middle planes of all 40× Z-stacks.

### Experimental Design and Statistical Analysis

This study is a between-subject design with respect to the current intensity used to induce seizures. Individual animals were randomly assigned to one of six current intensity treatment conditions (0, 20, 40, 65, 77 and 85 mA). Four brain regions were analyzed for each animal (DG granule cell expression, CA3 and CA1 pyramidal cell expression within the hippocampus, and pyramidal cell expression in area 35 of PRC). The main effect of differences in *Arc* expression as a result of ECT intensity for each hippocampal subregion was evaluated using either a one-way analysis of variance (ANOVA) followed by Bonferroni’s *post hoc* tests to evaluate *Arc* expression at varying current conditions within a region, or a two-way ANOVA to evaluate *Arc* expression at varying current conditions between brain regions. Alpha levels were all set at <0.05.

## Results

**Figure 1:**
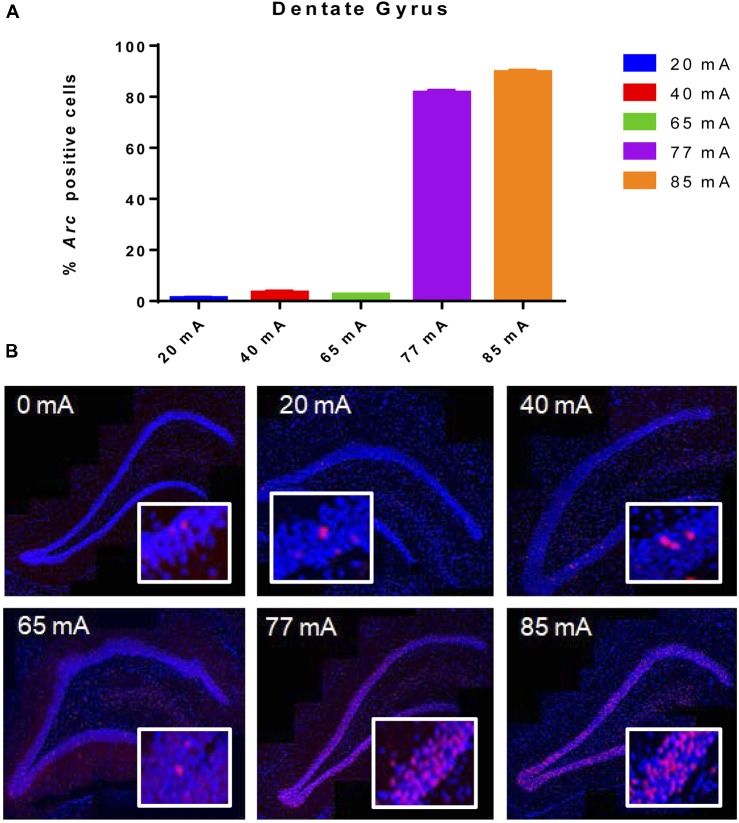
**(A)** *Arc* mRNA-positive cells in the dentate gyrus (DG) of animals that were given electroconvulsive shock at 20, 40, 65, 77 and 85 mA conditions. The proportion of Arc+ cells found in the caged-control condition was subtracted from the data in all current-intensity conditions. **(B)** Representative confocal images of DG from animals that were given electroconvulsive shock at 0, 20, 40, 65, 77 and 85 mA. Scale bar = 40 μm. Insets in each panel show high magnification of granule cells showing *Arc* labeling. Note that at intensities of 77 and 85 mA, most cells show transcriptional foci. Because of the cell packing density, there are many *Arc*-expressing cells within this higher magnification field of view.

Behavioral seizures were induced by all current intensities, resulting in hind-limb extension and tonic clonic motor responses. The 20, 40 or 65 mA conditions resulted in very low *Arc* mRNA expression in the DG (Figure 1). Excitation at 77 and 85 mA resulted in *Arc* mRNA expression in ~85 ± 1.0 and 92 ± 0.8% of granule cells. A one-way ANOVA showed a significant effect of current (*F*_(5,12)_ = 3327, *p* ≤ 0.001).

**Figure 2:**
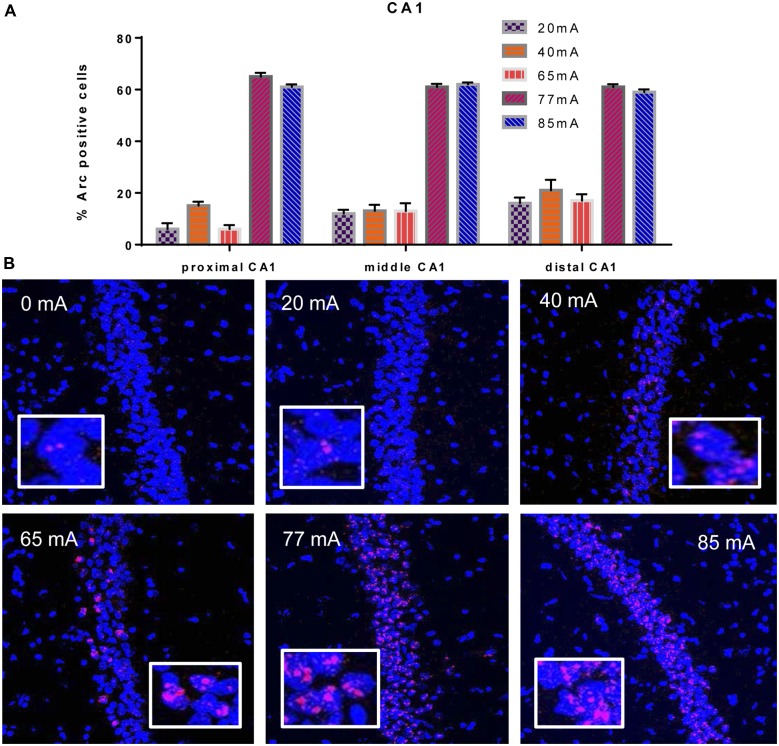
**(A)** *Arc* mRNA-positive CA1 pyramidal cells in the animals that were given electroconvulsive shock at 20, 40, 65, 77 and 85 mA conditions (proximal, middle and distal) are shown. The proportion of Arc+ cells found in the caged-control condition was subtracted from the data in all current-intensity conditions. **(B)** Representative confocal images of hippocampal CA1 sub-region from animals that were given electroconvulsive shock at 0, 20, 40, 65, 77 and 85 mA. Scale bar = 40 μm. Insets in each panel show high magnification of CA1 pyramidal cells showing *Arc* labeling.

We analyzed three different parts of CA1, Proximal CA1, Middle CA1 and Distal CA1, *Arc* mRNA positive pyramidal cells are shown in Figure 2. A two-way ANOVA revealed a significant effect of region (*F*_(2,42)_ = 3.9; *p* = 0.028) a significant effect of current (*F*_(5,42)_ = 305; *p* ≤ 0.001; *df* = 5), but no region by current interaction (*F*_(10,42)_ = 1.53; *p* = 0.16; *df* = 10). A repeated measures ANOVA showed an effect of region (*F*_(2,36)_ = 4.29; *p* = 0.025), but not a region by current interaction (*F*_(2,36)_ = 1.11; *p* = 0.40).

**Figure 3:**
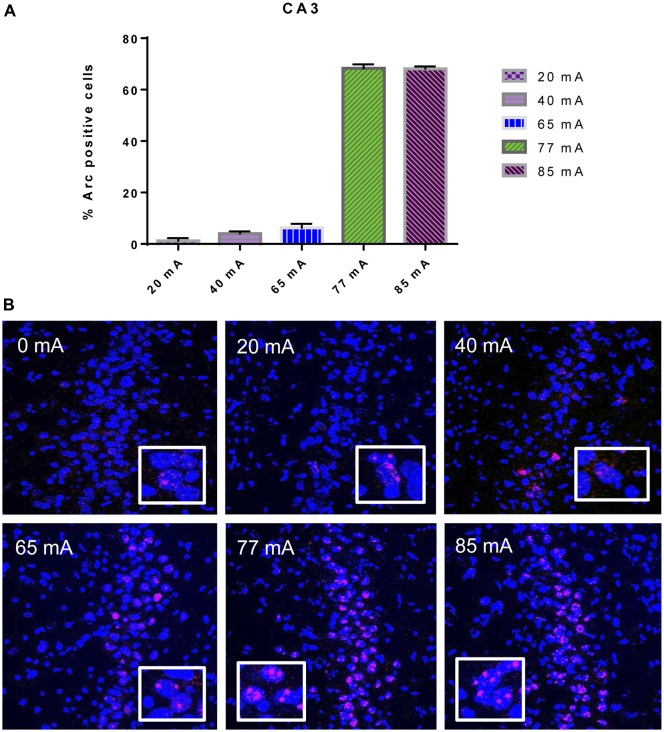
**(A)** *Arc* mRNA-positive pyramidal cells in the CA3 subregion of animals that were given electroconvulsive shock at 20, 40, 65, 77 and 85 mA. The proportion of Arc+ cells found in the caged-control condition was subtracted from the data in all current-intensity conditions. **(B)** Representative confocal images of hippocampal CA3 sub-region from animals that were given electroconvulsive shock at 0, 20, 40, 65, 77 and 85 mA. Scale bar = 40 μm. Insets in each panel show high magnification of CA3 pyramidal cells showing *Arc* labeling.

**Figure 4:**
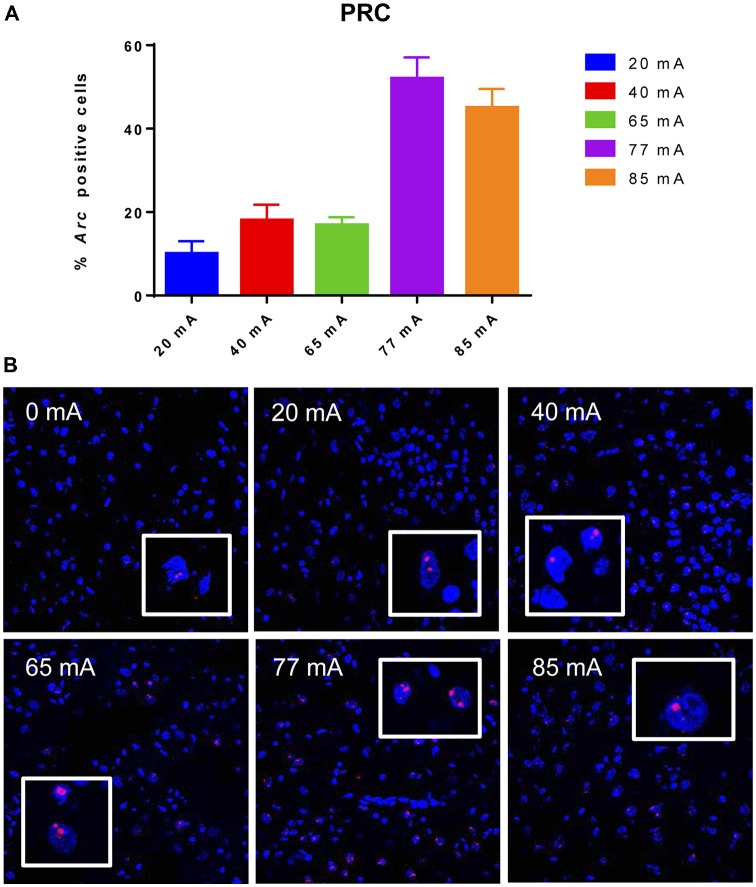
Panel **(A)** shows a graph of *Arc* mRNA positive principle cells in the perirhinal cortex (PRC) cortical region of animals that were given electroconvulsive shock at 20, 40, 65, 77 and 85 mA. The proportion of Arc+ cells found in the caged-control condition was subtracted from the data in all current-intensity conditions. **(B)** Representative confocal images of cortical PRC region from animals that were given electroconvulsive shock at 0, 20, 40, 65, 77 and 85 mA. Scale bar = 40 μm. Insets in each panel show high magnification of perirhinal cortical cells showing *Arc* labeling.

The *Arc* mRNA positive CA3 pyramidal cells are shown in Figure 3. A one-way ANOVA revealed a significant effect of current (*F*_(5,14)_ = 445.8, *p* ≤ 0.001). The *Arc* mRNA positive PRC principle cells are shown in Figure 4. A one-way ANOVA showed a significant effect of current (*F*_(5,12)_ = 34.06, *p* ≤ 0.001).

**Figure 5:**
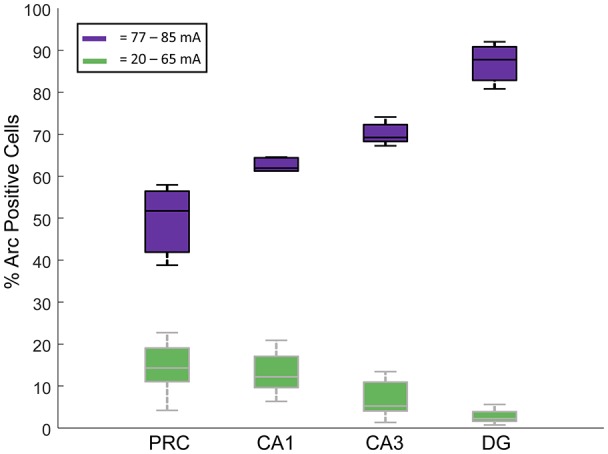
Proportions of *Arc* mRNA-positive cells in each of the four regions were examined (DG, CA1, CA3 and PRC). In green are the % *Arc*-positive cells in rats given electroconvulsive shock treatment (ECT) at stimulus intensities between 20 mA and 65 mA. In purple are the % *Arc*-positive cells in rats given ECT at stimulus intensities of 77–85 mA. All data were normalized by subtracting the % *Arc*-positive cells from caged control rats. Note that at the lowest current amplitudes, the DG showed the fewest *Arc*-positive cells, followed by CA3, then CA1 and finally PRC. At the higher current intensities, however, the opposite pattern of activation was observed.

Interestingly, when we compared the proportions of cells that showed *Arc* expression in each cell type across the four regions examined, separated by current intensities below and above the apparent threshold current of  77 mA, we noted that the regions with the fewest *Arc*-expressing cells below the apparent threshold had more *Arc*-expressing cells above this threshold (two-way ANOVA, Region: *F*_(3,54)_ = 17.61, *p* < 0.001; Threshold: *F*_(1,54)_ = 2269, *p* < 0.001; Region * Threshold Interaction: *F*_(3,54)_ = 73.81, *p* < 0.001; Figure 5). Also see Table 1 for the caged control Arc mRNA positive cells in all the subregions examined.

**Table 1:** *Arc* mRNA expression in cage controls.

Region	% *Arc* mRNA positive cells
Dentate gyrus	cage control = 1 ± 1.7
CA1 (proximal)	cage control = 8 ± 0.3
CA1 (middle)	cage control = 10 ± 1.8
CA1 (distal)	cage control = 9 ± 1.9
CA3	cage control = 5 ± 1.0
Perirhinal cortex	cage control = 14 ± 1.7

## Discussion

These results provide evidence for the first time that there is a threshold level of depolarization that must be crossed before *Arc* mRNA transcription occurs following ECT. Even at the lowest ECT current (20 mA), all rats exhibited the behavioral manifestation of seizures, yet the sharp intensity threshold for the cellular expression of *Arc* did not follow the behavioral pattern. Rather, cells in the DG, CA1, CA3 and PRC required higher intensities for the full transcriptional response to be triggered. Even though there were regional differences in the absolute proportion of cells that expressed *Arc* at both lower (20, 40 and 65 mA), and higher (77 and 85 mA), current intensities, all regions showed the same pattern of abrupt, near maximal expression at 77 mA.

### Regional Differences in the Numbers of *Arc* mRNA-Positive Cells Following Seizure Stimulation

An interesting interregional difference noted in the present experiment was that the areas that were most responsive to the lowest stimulus intensities were also the areas that showed the greatest proportion of *Arc*-positive cells at stimulus currents above the 77 mA threshold (Figure 5). Specifically, the DG had the most *Arc*-positive neurons activated at the higher current conditions, followed by CA3, and then CA1. The exact opposite pattern was observed following the lowest stimulus currents—CA1 showed the greatest numbers of *Arc*-expressing cells, followed by CA3 and then DG. One possible explanation for the observation that DG granule cells are the least responsive to the lower stimulus intensities is that these cells tend to be under greater inhibitory control than are hippocampal pyramidal cells (e.g., Acsády and Káli, 2007). The fact that the granule cells show the greatest proportion of *Arc*-expressing cells following delivery of the highest stimulus intensities may be understood in terms of the recurrent excitatory nature of the CA3-DG network (Ribak et al., 1985; Claiborne et al., 1986; Ishizuka et al., 1990; Li et al., 1994; Acsády et al., 1998). These excitatory connections may serve to amplify local network responses to ECT once a certain stimulation intensity is surpassed, resulting in more activated neurons in this region.

### Implications for Therapeutics Involving Electroconvulsive Shock Treatment

Many therapeutic effects that result from ECT are thought to arise from the altered expression of various activity-dependent target genes that encode transcription factors, structural proteins, and neuropeptides. The present observation that the full IEG response is not activated below a specific stimulation strength suggests that some of the therapeutic benefits of ECT treatment cannot be achieved without the appropriate stimulation intensity. If different genes do have particular activation thresholds, then it is possible that ECT delivered at different intensity settings could result in unique gene-activation profiles that may result in distinct therapeutic effects. Whether other ECT target genes show similar induction thresholds as *Arc* remains to be investigated systematically, and this is an important question for understanding the mechanisms giving rise to the therapeutic benefits of ECT.

### Dendritic Ca^2+^ Potentials, Burst-Firing and the Threshold for *Arc* Activation

It is well-established that IEG mRNA transcription is initiated following electrical stimulation that results in seizures (Morgan et al., 1987). Focal, unilateral injections of tetrodotoxin (TTX) into the hippocampus prior to seizure induction results in reduced mRNA expression only in the TTX-injected side, suggesting a role for spiking activity in the ECT-induced mRNA response. Convergent afferent stimulation can result in dendritic Ca^2+^ potentials in hippocampal CA1 neurons that summate supralinearly to create relatively large dendritic Ca^2+^ plateau potentials (Takahashi and Magee, 2009; Bittner et al., 2015). For example coincident stimulation of Schaffer collateral and perforant path inputs onto CA1 pyramidal cells results in these large potentials, whereas the stimulation of either projection in isolation does not (Takahashi and Magee, 2009). These plateau potentials appear to drive burst firing *in vivo* (Apostolides et al., 2016), which is a physiological pattern of activity thought to be critical for mechanisms of behavior-driven place field expression (O’Keefe, 1976), and spike timing-dependent plasticity (Bi and Poo, 1998). Moreover, *in vivo* intracellular recordings from CA1 pyramidal neurons suggest that a number of voltage-gated Ca^2+^ channels (e.g., Grienberger et al., 2014) are likely to participate in the initiation of these dendritic potentials.

Whether Ca^2+^ plateau potentials can directly regulate *Arc* mRNA expression *in vivo* has not been shown directly. During exploratory behavior, however, place-specific burst firing is known to arise in the hippocampus *only* after these potentials emerge—as though the plateau potentials serve to prime different neurons within an ensemble to develop feature-specific firing patterns (Bittner et al., 2015). Remarkably, experimentally-induced dendritic plateau potentials result in place cell activity at that particular stimulation location on subsequent traversals of a virtual reality track (Bittner et al., 2015). Hippocampal *Arc* transcription is induced in a context-specific manner that quantitatively and qualitatively resembles ensemble activity recorded in electrophysiological studies (Guzowski et al., 2004, 2006; Kubik et al., 2007), and its expression is known to be Ca^2+^-dependent (Bramham et al., 2008; Nikolaienko et al., 2018). These studies suggest that the recruitment of *Arc* transcriptional responses could similarly require a threshold level of depolarization, which is clearly reflected in all regions examined in the present study. Whether mechanisms involved in producing these Ca^2+^ plateau potentials are the only explanation for the findings in the present study remains to be investigated.

### What Is Responsible for the Transcriptional Regulation of the Arc Gene?

Given the strong evidence that the Arc gene requires a specific level of depolarization in any given cell before transcription can be initiated, a number of possible mechanisms can be suggested. For example, epigenetic processes are known to be involved in regulating transcription in an activity-dependent manner (e.g., Miller and Sweatt, 2007; Nagy et al., 2007). In aging rats, fewer granule cells express *Arc* mRNA following behavior (using catFISH methodology) while in the same animals the numbers of pyramidal cells in CA3 and CA1 that express *Arc* do not differ (Small et al., 2004). Penner et al. (2011) replicated this finding, and showed that, in fact, old DG granule cells show reduced exploration-induced *Arc* transcription as measured by qPCR (about half as much as do adult granule cells). Furthermore, there was increased methylation of the Arc gene in old granule cells, suggesting that these cells are held in a state less conducive to transcription. This raises the possibility that regulation of the methylation state of the Arc gene could be one mechanism by which transcriptional thresholds are implemented. It remains to be determined, however, if methylation levels are reduced in parallel with Arc transcription thresholds, or potentially by other epigenetic processes.

## Author Contributions

MC performed brain extractions, sectioning, *in situ* hybridizations, confocal microscopy and cell counting, performed analysis and manuscript preparation. DG assisted in MECS for the threshold experiment, statistical analysis and manuscript preparation. CN assisted with confocal microscopy and cell counting. HD assisted with confocal microscopy and cell counting. MH, HO and CB developed the concept. CB and MZ performed MES for the behavior assessment and edited the manuscript.

## Conflict of Interest Statement

The authors declare that the research was conducted in the absence of any commercial or financial relationships that could be construed as a potential conflict of interest.

## References

[B1] Acsády L. Káli S. (2007). “Models, structure, function: the transformation of cortical signals in the dentate gyrus,” in Progress in Brain Research The Dentate Gyrus: A Comprehensive Guide to Structure, Function and Clinical Implications, ed. Scharfman H. E. (Amsterdam: Elsevier), 577–599. 10.1016/S0079-6123(07)63031-3 17765739

[B2] Acsády L. Kamondi A. Sík A. Freund T. Buzsáki G. (1998). GABAergic cells are the major postsynaptic targets of mossy fibers in the rat hippocampus. J. Neurosci. 18, 3386–3403. 10.1523/jneurosci.18-11-j0001.1998 9547246 PMC6792657

[B3] Altar C. A. Whitehead R. E. Chen R. Wörtwein G. Madsen T. M. (2003). Effects of electroconvulsive seizures and antidepressant drugs on brain-derived neurotrophic factor protein in rat brain. Biol. Psychiatry 54, 703–709. 10.1016/s0006-3223(03)00073-8 14512210

[B100] Apostolides P. F. Milstein A. D. Grienberger C. Bittner K. C. Magee J. C. (2016). Axonal filtering allows reliable output during dendritic plateau-driven complex spiking in CA1 neurons. Neuron 89, 770–783. 10.1016/j.neuron.2015.12.040 26833135

[B4] Bi G. Q. Poo M. M. (1998). Synaptic modifications in cultured hippocampal neurons: dependence on spike timing, synaptic strength and postsynaptic cell type. J. Neurosci. 18, 10464–10472. 10.1523/jneurosci.18-24-10464.1998 9852584 PMC6793365

[B5] Bittner K. C. Grienberger C. Vaidya S. P. Milstein A. D. Macklin J. J. Suh J.. (2015). Conjunctive input processing drives feature selectivity in hippocampal CA1 neurons. Nat. Neurosci. 18, 1133–1142. 10.1038/nn.4062 26167906 PMC4888374

[B7] Bramham C. R. Worley P. F. Moore M. J. Guzowski J. F. (2008). The immediate early gene Arc/Arg3.1: regulation, mechanisms, and function. J. Neurosci. 28, 11760–11767. 10.1523/jneurosci.3864-08.2008 19005037 PMC2615463

[B8] Brunoni A. R. Baeken C. Machado-Vieira R. Gattaz W. F. Vanderhasselt M.-A. (2014). BDNF blood levels after electroconvulsive therapy in patients with mood disorders: a systematic review and meta-analysis. World J. Biol. Psychiatry 15, 411–418. 10.3109/15622975.2014.892633 24628093

[B9] Chawla M. K. Guzowski J. F. Ramirez-Amaya V. Lipa P. Hoffman K. L. Marriott L. K.. (2005). Sparse, environmentally selective expression of arc RNA in the upper blade of the rodent fascia dentata by brief spatial experience. Hippocampus 15, 579–586. 10.1002/hipo.20091 15920719

[B10] Chawla M. K. Sutherland V. L. Olson K. McNaughton B. L. Barnes C. A. (2018). Behavior-driven arc expression is reduced in all ventral hippocampal subfields compared to CA1, CA3 and dentate gyrus in rat dorsal hippocampus. Hippocampus 28, 178–185. 10.1002/hipo.22820 29232477 PMC5777901

[B11] Chowdhury S. Shepherd J. D. Okuno H. Lyford G. Petralia R. S. Plath N.. (2006). Arc/Arg3.1 interacts with the endocytic machinery to regulate AMPA receptor trafficking. Neuron 52, 445–459. 10.1016/j.neuron.2006.08.033 17088211 PMC1784006

[B12] Claiborne B. J. Amaral D. G. Cowan W. M. (1986). A light and electron microscopic analysis of the mossy fibers of the rat dentate gyrus. J. Comp. Neurol. 246, 435–458. 10.1002/cne.902460403 3700723

[B13] Dolmetsch R. E. Pajvani U. Fife K. Spotts J. M. Greenberg M. E. (2001). Signaling to the nucleus by an L-type calcium channel-calmodulin complex through the MAP kinase pathway. Science 294, 333–339. 10.1126/science.1063395 11598293

[B102] Grienberger C. Chen X. Konnerth A. (2014). NMDA receptor-dependent multidendrite Ca^2+^ spikes required for hippocampal burst firing in vivo. Neuron 81, 1274–1281. 10.1016/j.neuron.2014.01.014 24560703

[B14] Guzowski J. F. McNaughton B. L. Barnes C. A. Worley P. F. (1999). Environment-specific expression of the immediate-early gene arc in hippocampal neuronal ensembles. Nat. Neurosci. 2, 1120–1124. 10.1038/16046 10570490

[B15] Guzowski J. F. McNaughton B. L. Barnes C. A. Worley P. F. (2001). Imaging neural activity with temporal and cellular resolution using FISH. Curr. Opin. Neurobiol. 11, 579–584. 10.1016/s0959-4388(00)00252-x 11595491

[B103] Guzowski J. F. Knierim J. J. Moser E. I. (2004). Ensemble dynamics of hippocampal regions CA3 and CA1. Neuron 44, 581–584. 10.1016/j.neuron.2004.11.003 15541306

[B104] Guzowski J. F. Miyashita T. Chawla M. K. Sanderson J. Maes L. I. Houston F. P.. (2006). Recent behavioral history modifies coupling between cell activity and *Arc* gene transcription in hippocampal CA1 neurons. Proc. Natl. Acad. Sci. U S A 103, 1077–1082. 10.1073/pnas.0505519103 16415163 PMC1347968

[B16] Hartzell A. L. Burke S. N. Hoang L. T. Lister J. P. Rodriguez C. N. Barnes C. A. (2013). Transcription of the immediate-early gene arc in CA1 of the hippocampus reveals activity differences along the proximodistal axis that are attenuated by advanced age. J. Neurosci. 33, 3424–3433. 10.1523/jneurosci.4727-12.2013 23426670 PMC3711759

[B17] Hu Y. Yu X. Yang F. Si T. Wang W. Tan Y.. (2010). The level of serum brain-derived neurotrophic factor is associated with the therapeutic efficacy of modified electroconvulsive therapy in Chinese patients with depression. J. ECT 26, 121–125. 10.1097/yct.0b013e3181c18bbf 19935088

[B18] Ishizuka N. Weber J. Amaral D. G. (1990). Organization of intrahippocampal projections originating from CA3 pyramidal cells in the rat. J. Comp. Neurol. 295, 580–623. 10.1002/cne.902950407 2358523

[B19] Jarsky T. Roxin A. Kath W. L. Spruston N. (2005). Conditional dendritic spike propagation following distal synaptic activation of hippocampal CA1 pyramidal neurons. Nat. Neurosci. 8, 1667–1676. 10.1038/nn1599 16299501

[B20] Kamondi A. Acsády L. Buzsáki G. (1998). Dendritic spikes are enhanced by cooperative network activity in the intact hippocampus. J. Neurosci. 18, 3919–3928. 10.1523/jneurosci.18-10-03919.1998 9570819 PMC6793142

[B105] Kubik S. Miyashita T. Guzowski J. F. (2007). Using immediate-early genes to map hippocampal subregional functions. Learn. Mem. 14, 758–770. 10.1101/lm.698107 18007019

[B21] Leal G. Bramham C. R. Duarte C. B. (2017). BDNF and hippocampal synaptic plasticity. Vitam. Horm. 104, 153–195. 10.1016/bs.vh.2016.10.004 28215294

[B22] Li X.-G. Somogyi P. Ylinen A. Buzsáki G. (1994). The hippocampal CA3 network: an *in vivo* intracellular labeling study. J. Comp. Neurol. 339, 181–208. 10.1002/cne.903390204 8300905

[B23] Lu B. Nagappan G. Lu Y. (2014). BDNF and synaptic plasticity, cognitive function and dysfunction. Handb. Exp. Pharmacol. 220, 223–250. 10.1007/978-3-642-45106-5_9 24668475

[B24] Lyford G. L. Yamagata K. Kaufmann W. E. Barnes C. A. Sanders L. K. Copeland N. G.. (1995). Arc, a growth factor and activity-regulated gene, encodes a novel cytoskeleton-associated protein that is enriched in neuronal dendrites. Neuron 14, 433–445. 10.1016/0896-6273(95)90299-6 7857651

[B106] Miller C. A. Sweatt J. D. (2007). Covalent modification of DNA regulates memory formation. Neuron 53, 857–869. 10.1016/j.neuron.2007.02.022 17359920

[B25] Morgan J. I. Cohen D. R. Hempstead J. L. Curran T. (1987). Mapping patterns of c-fos expression in the central nervous system after seizure. Science 237, 192–197. 10.1126/science.3037702 3037702

[B107] Nagy A. Gertsenstein M. Vintersten K. Behringer R. (2007). Preparing glass slides and coverslips for *in situ* hybridization. Cold Spring Harb. Protoc. 2007:pdb.prot4817. 10.1101/pdb.prot4817 21356972

[B26] Nibuya M. Sugiyama H. Shioda K. Nakamura K. Nishijima K. (2002). ECT for the treatment of psychiatric symptoms in basedow’s disease. J. ECT 18, 54–57. 10.1097/00124509-200203000-00014 11925523

[B27] Nikolaienko O. Patil S. Eriksen M. S. Bramham C. R. (2018). Arc protein: a flexible hub for synaptic plasticity and cognition. Semin. Cell Dev. Biol. 77, 33–42. 10.1016/j.semcdb.2017.09.006 28890419

[B108] O’Keefe J. (1976). Place units in the hippocampus of the freely moving rat. Exp. Neurol. 51, 78–109. 10.1016/0014-4886(76)90055-8 1261644

[B28] Okuno H. Akashi K. Ishii Y. Yagishita-Kyo N. Suzuki K. Nonaka M.. (2012). Inverse synaptic tagging of inactive synapses via dynamic interaction of Arc/Arg3.1 with CaMKIIβ. Cell 149, 886–898. 10.1016/j.cell.2012.02.062 22579289 PMC4856149

[B109] Penner M. R. Roth T. L. Chawla M. K. Hoang L. T. Roth E. D. Lubin F. D.. (2011). Age-related changes in *Arc* transcription and DNA methylation within the hippocampus. Neurobiol. Aging 32, 2198–2210. 10.1016/j.neurobiolaging.2010.01.009 20189687 PMC2888808

[B29] Park H. Poo M. M. (2013). Neurotrophin regulation of neural circuit development and function. Nat. Rev. Neurosci. 14, 7–23. 10.1038/nrn3379 23254191

[B110] Ramírez-Amaya V. Vazdarjanova A. Mikhael D. Rosi S. Worley P. F. Barnes C. A. (2005). Spatial exploration-induced *Arc* mRNA and protein expression: evidence for selective, network-specific reactivation. J. Neurosci. 25, 1761–1768. 10.1523/jneurosci.4342-04.2005 15716412 PMC6725922

[B30] Ribak C. E. Seress L. Amaral D. G. (1985). The development, ultrastructure and synaptic connections of the mossy cells of the dentate gyrus. J. Neurocytol. 14, 835–857. 10.1007/bf01170832 2419523

[B31] Shepherd J. D. Rumbaugh G. Wu J. Chowdhury S. Plath N. Kuhl D.. (2006). Arc/Arg3.1 mediates homeostatic synaptic scaling of AMPA receptors. Neuron 52, 475–484. 10.1016/j.neuron.2006.08.034 17088213 PMC1764219

[B32] Singh A. Kar S. K. (2017). How electroconvulsive therapy works?: understanding the neurobiological mechanisms. Clin. Psychopharmacol. Neurosci. 15, 210–221. 10.9758/cpn.2017.15.3.210 28783929 PMC5565084

[B33] Sjöström P. J. Häusser M. (2006). A cooperative switch determines the sign of synaptic plasticity in distal dendrites of neocortical pyramidal neurons. Neuron 51, 227–238. 10.1016/j.neuron.2006.06.017 16846857 PMC7616902

[B111] Small S. A. Chawla M. K. Buonocore M. Rapp P. R. Barnes C. A. (2004). Imaging correlates of brain function in monkeys and rats isolates a hippocampal subregion differentially vulnerable to aging. Proc. Natl. Acad. Sci. U S A 101, 7181–7186. 10.1073/pnas.0400285101 15118105 PMC406486

[B34] Tabuchi A. Nakaoka R. Amano K. Yukimine M. Andoh T. Kuraishi Y.. (2000). Differential activation of brain-derived neurotrophic factor gene promoters I and III by Ca^2+^ signals evoked vial-type voltage-dependent and n-methyl-d-aspartate receptor Ca^2+^ channels. J. Biol. Chem. 275, 17269–17275. 10.1074/jbc.m909538199 10748141

[B35] Takahashi H. Magee J. C. (2009). Pathway interactions and synaptic plasticity in the dendritic tuft regions of CA1 pyramidal neurons. Neuron 62, 102–111. 10.1016/j.neuron.2009.03.007 19376070

[B36] Takasu M. A. Dalva M. B. Zigmond R. E. Greenberg M. E. (2002). Modulation of NMDA receptor-dependent calcium influx and gene expression through EphB receptors. Science 295, 491–495. 10.1126/science.1065983 11799243

[B37] Tsay D. Dudman J. T. Siegelbaum S. A. (2007). HCN1 channels constrain synaptically evoked Ca^2+^ spikes in distal dendrites of CA1 pyramidal neurons. Neuron 56, 1076–1089. 10.1016/j.neuron.2007.11.015 18093528 PMC2435011

[B38] Wallace C. S. Lyford G. L. Worley P. F. Steward O. (1998). Differential intracellular sorting of immediate early gene mRNAs depends on signals in the mRNA sequence. J. Neurosci. 18, 26–35. 10.1523/jneurosci.18-01-00026.1998 9412483 PMC6793378

[B39] Zhang X. Zhang Z. Sha W. Xie C. Xi G. Zhou H.. (2009). Electroconvulsive therapy increases glial cell-line derived neurotrophic factor (GDNF) serum levels in patients with drug-resistant depression. Psychiatry Res. 170, 273–275. 10.1016/j.psychres.2009.01.011 19896212

